# Measurement of Canine Ataxic Gait Patterns Using Body-Worn Smartphone Sensor Data

**DOI:** 10.3389/fvets.2022.912253

**Published:** 2022-08-04

**Authors:** Daniel Engelsman, Tamara Sherif, Sebastian Meller, Friederike Twele, Itzik Klein, Anna Zamansky, Holger A. Volk

**Affiliations:** ^1^The Hatter Department of Marine Technologies, University of Haifa, Haifa, Israel; ^2^Department of Small Animal Medicine and Surgery, University of Veterinary Medicine Hanover, Hanover, Germany; ^3^Information Systems Department, University of Haifa, Haifa, Israel; ^4^Center for Systems Neuroscience, Hanover, Germany

**Keywords:** ataxia, inertial measurement unit (IMU), smartphone and IoT services, neurology, canis, gait analysis, wearable and mobile computing

## Abstract

Ataxia is an impairment of the coordination of movement or the interaction of associated muscles, accompanied by a disturbance of the gait pattern. Diagnosis of this clinical sign, and evaluation of its severity is usually done using subjective scales during neurological examination. In this exploratory study we investigated if inertial sensors in a smart phone (3 axes of accelerometer and 3 axes of gyroscope) can be used to detect ataxia. The setting involved inertial sensor data collected by smartphone placed on the dog's back while walking in a straight line. A total of 770 walking sessions were evaluated comparing the gait of 55 healthy dogs to the one of 23 dogs with ataxia. Different machine learning techniques were used with the K-nearest neighbors technique reaching 95% accuracy in discriminating between a healthy control group and ataxic dogs, indicating potential use for smartphone apps for canine ataxia diagnosis and monitoring of treatment effect.

## 1. Introduction

Neurological disorders are among the most severe and difficult-to-treat pathological conditions in human and veterinary medicine. In a random sample of dogs attending 89 clinics in the United Kingdom between 2009 and 2013, every 10th patient was diagnosed with a neurological disorder (1). Often, they progress with no prospect of cure and diagnosis and treatment occurring too late to make a difference, ending only with euthanasia (2, 3). Ataxia dramatically impairs the quality of life not only of the affected animals but also of their caretakers (4). Ataxia is a greek term describing a ‘lack of order'. Canine ataxia is defined as an impairment of the coordination of movement or the interaction of associated muscles and is accompanied by a disturbance of the gait pattern (5). Since ataxia is a clinical sign and not a disease (5), a precise assessment of the neuroanatomical localization of the lesion causing the gait disorder is necessary for appropriate diagnosis and therapy (6, 7). Ataxia is a sensory phenomenon causing the aforementioned disturbance of coordination of movement, and may occur as a sensory (proprioceptive), cerebellar or vestibular ataxia (or a combination of these) (6, 8–10). Sensory ataxia causes a loss of sense of limb and body position, often seen as wide-based stance, swaying gait, increased (Upper Motor Neuron, UMN) or decreased (Lower Motor Neuron, LMN) stride length, and dragging or scuffing of the digits. It is caused by a lesion of the afferent sensory (proprioceptive) pathways in the peripheral nerves or centrally in the spinal cord, brainstem or forebrain. Cerebellar ataxia (11) is characterized by an inability to control the rate and range of movement, truncal swaying, resulting in dysmetria (often hypermetria), and intention tremor. Cerebellar ataxia occurs with lesions of the cerebellum or spinocerebellar tracts within the spinal cord. Vestibular ataxia is seen as leaning, falling or rolling to one side. Head tilt and abnormal nystagmus may be present. Vestibular ataxia results from lesions of the vestibular system peripherally (receptors in the inner ear, vestibulocochlear nerve) or centrally (brainstem, cerebellum).

These types of ataxia and their associated clinical signs are usually recognized during a neurological examination and subjectively assessed. However, manual assessment by experts has serious limitations. Regardless of the degree of the observer's experience, the human eye is not capable of processing the full complexity of all components of the movement patterns (12). In dogs, it has been also shown that obvious irregularities in gait, such as orthopedically caused lameness, cannot reliably be quantified when observed by experienced orthopedic surgeons (13, 14). Ataxia in animals can also be difficult to quantify with poor interobserver agreement, despite using raters with high clinical competence levels [see, e.g., Olsen et al. (15) for horses]. Thus, there is the urgent need to use objective gait analysis systems, especially when quantifying levels of ataxia.

An objective gait assessment would be an essential tool for clinics not only to diagnose different neurological conditions but also to monitor disease progression and therapeutic effects without human biases. There are well-established methods and models for data collection and interpretation within different pathologies in people (16), highlighting that instrumentation of gait using digital technologies provides objective and subtle information that is not possible to detect from clinical observation alone. For quadrupeds, objective assessment of ataxia gait patterns was studied in Olsen et al. (17). It was found that motion capture can objectively aid the assessment of horses with ataxia; moreover, blindfolding of horses facilitates the discrimination of ataxic patterns.

**Figure 1:**
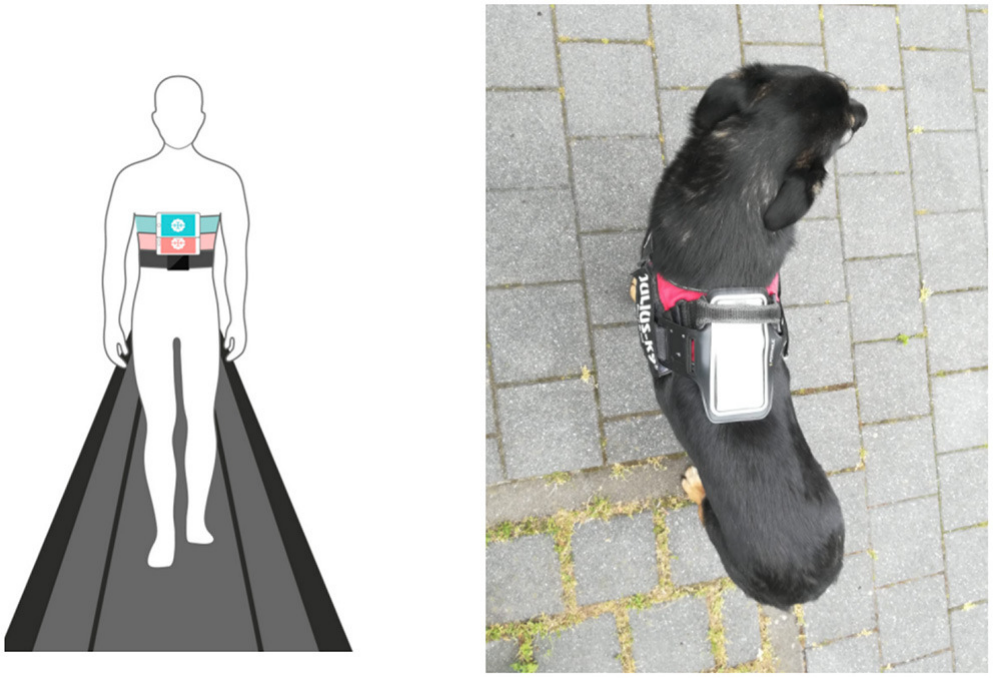
**Left:** placement of smartphone in Encephalog (Mon4t) iTUG test strapped to the subject's sternum; **Right:** placement of smartphone strapped to the dog's back in our study.

Smartphone inertial sensors are commonly used in human activity recognition tasks (18, 19), as well as smartphone location recognition (20), both essential for human gait analysis as part of pedestrian dead reckoning application (21, 22). Yahalom et al. (23) and Tchelet et al. (24) used the EncephaLogTM (Mon4t) platform for collecting internal motion data for conducting motor evaluation in the context of human neurology, which utilizes smartphones' internal motion unit (IMU) sensors. It was validated against motion capture cameras, pressure mat and wearable sensors used in motion labs in instrumented Completion-time of the Timed-Up-and-Go (TUG) test, a well-accepted clinical biomarker for rating mobility and prediction of falls risk. TUG Completion Time and nine additional biomarkers were validated against in Yahalom et al. (23) and Tchelet et al. (24) respectively, suggesting that EncephaLog (Mon4t) can provide an accurate, yet simpler, instrumented TUG platform than existing alternatives, offering a solution for clinics that cannot afford the cost or space required for a dedicated motion lab and for monitoring patients at their homes. In the iTUG test the smartphone device running EncephaLog is strapped to the subject's sternum. In the current study, adapting the data collection protocol to dogs, we placed the smartphone device on the dog's back, securing it with an adjustable harness and bracket, as shown in Figure 1.

Gait analysis of dogs is commonly performed using pressure walkways (25), force plates (26), treadmills (27), computer-assisted motion capture (28), and kinematic gait analysis (29). These methods require expensive equipment to collect and analyze data and are time-intensive. They are therefore rarely used as a day-to-day clinical tool. Thus, body-worn devices may provide an attractive simpler and cheaper alternative. Yet, in contrast to the human domain, canine gait analysis using body-worn devices remains underexplored.

To close this scientific gap, the current study investigated whether IMU data obtained from a body-worn smartphone can be used to automatically classify between dogs diagnosed with ataxia, and a healthy control group.

## 2. Methods

Data collection was performed at the Department for Small Animal Medicine and Surgery of the University of Veterinary Medicine Hannover, Germany. The Lower Saxony State Office for Consumer Protection and Food Safety in Oldenburg, Germany has approved the study (reference number for this project: 20A555). Owners of participating dogs received a fact sheet prior to the day of data collection and were informed about the procedure in a personal conversation. All owners provided written consent for study participation, as well as signed a form about our privacy and data protection policy.

Prior to objective gait assessment patient history evaluation was performed and during general examination orthopedic diseases were excluded in order to ensure only neurologically caused gait incoordination were included. In addition, neurological examination was performed by board certified diplomates as well as residents of the European College of Veterinary Neurology (ECVN). They agreed upon a neuroanatomical localization of the lesion and differentiated the aforementioned forms of ataxia. During data collection the dogs simultaneously underwent continuous inspection by the same investigator and any gait abnormalities were recorded.

The smartphone used for gait assessment was an iPhone SE (Apple Inc.) on which a customized version of the EncephalogClinic^®^ (Mon4t) app was installed. Modern smartphones contain an electronic device called IMU containing two types of inertial sensors—accelerometers and gyroscopes (30) the recordings of which are captured by the EncephalogClinic^®^ (Mon4t) app upon its actication. An accelerometer measures the specific force vector and a gyroscope measures the angular rate vector. In the collected dataset, a single data point is a pair of three-dimensional accelerometer and gyroscopes readings. The duration of a single sample (several data points) is determined by a fixed window size (WS), thus, the data signal can be viewed as a multivariate time series, and we apply standard techniques of feature extraction and feature selection (31) to it, as illustrated in **Figure 3**.

**Figure 2:**
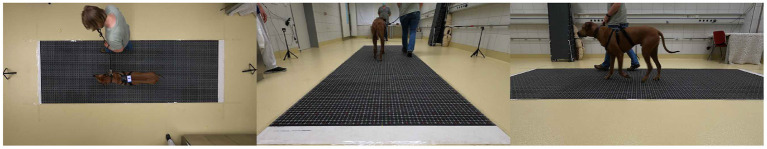
Phone placed on back of the dog using harness; photos from different sides.

The smartphone was fixed on the dog's body using an adjustable elastic harness (Julius-K9^®^, Hungary) with a bracket on top in which the smartphone was securely placed on the participant's back, as shown in Figure 2.

An individual adaptation phase to the gait analysis laboratory as well as the surrounding technical equipment and the harness with the smartphone on the dogs' dorsum was implemented in order to guarantee a smooth and relaxed, most possibly natural walking. Participating dogs were then led on a 150 cm long, loose leash to walk back and forth on a 140 x 400 cm, skid resistant felt grid mat with regularly arranged crisscross marks. Walking was performed in a slow pace in a straight line in the center of the grid mat. While orthopedic disease usually causes more severely affected gait abnormalities when the pace is increased, the opposite is true for ataxia. The integration of movement is best observed in slower pace. Observations at a slow walking pace for gait evaluation in neurological disorders ensure that the clinician does not miss neurological gait deficits. Walking pace was selected by the dog and only artificially corrected by the owner if the dog started trotting.

This protocol was repeated for at least five times, in order to reach at least a minimum of 50 strides, which is based on the experience from our former studies into ataxia evaluation (32, 33).

To be able to retrospectively reconstruct each gait assessment session the procedure was filmed from five perspectives (each side and bird's eye view).

Walking sessions were performed during routine neurological examination and could be easily implemented to clinical engagement. Owners of participating dogs were informed prior to the procedure and provided written consent.

## 3. Results

**Figure 3:**
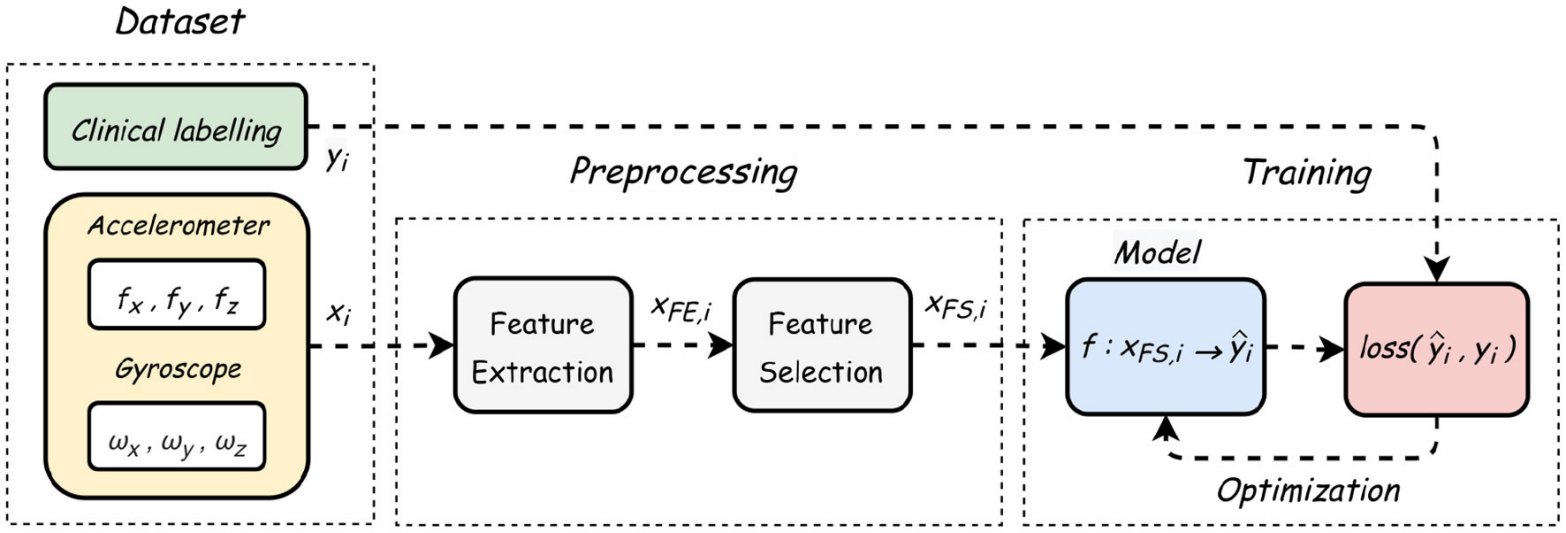
Flowchart diagram showing the process from data collection to model training.

This section presents the functional flow of a sample signal starting from dataset, through preprocessing, ending with model evaluation, as demonstrated in Figure 3.

### 3.1. Data Collection

Data was collected from 507 walking sessions of 55 healthy control group dogs (39 females and 16 males) and samples from 263 walking sessions of 23 dogs with ataxia (9 females and 14 males). Median age of dogs was 3 years (range 1–12 years) and 6 years (range 1–13 years) for the control group and the ataxic group, respectively. Median weight of dogs was 15.5 kg (range 7–32 kg) and 20 kg (range 4.4–41.4 kg) for the control group and the ataxic group, respectively. Ataxia was caused by spinal cord lesions in 11 dogs, by cerebellar disorders in 4 dogs, and 8 dogs had ataxia secondary to phenobarbitone treatment. Ataxia of one dog was of uncertain origin. The patient data is included in the Supplementary Material.

The recordings were made by the Mon4t app at a sampling rate of 100Hz; the Mon4t company made them available for us for downloading. Preprocessing, data analysis and modeling were performed using Python. Supplementary Figure 1 presents two different samples of healthy (blue) and ataxic (red) dogs, measured by the accelerometer (left) and the gyroscopes (right).

#### 3.1.1. Data Preparation

Since the samples had stagnant “tails” at the beginning and end, where the dog was recorded prior to walking start (when the tester pushed the start button on the app), the following cleaning procedure was conducted to guarantee that a given time window captures only gait: (i) Minimum acceleration threshold, to remove stationary segments. (ii) K-means clustering, to remove further outliers.

Next, an heuristic search was performed over the original dataset, to examine the influence of a given sample length, namely window size (WS), over the accuracy of a trained model. Using a logistic regression (LR) model, Supplementary Table 1 shows how an optimal window size could be determined, since its duration affects the number of available samples for each class. The tradeoff is evident: the longer the WS, the slower the periodicities that can be captured; in contrast, shorter WS yields more samples from each raw measurement, thus improving the model generalizability. As can be seen below, different WS elicits different datasets, characterized by a different number of class samples and a different Healthy/Ataxic ratio. Next, each dataset is evaluated by the LR model, to allow an empirical benchmarking, following the traditional 80:20 train-test split ratio. As marked in the table, the optimal window size was chosen to be three seconds, with a total of 1210 samples.

#### 3.1.2. Feature Engineering

To further evaluate machine learning approaches, 15 different statistical and frequency features, presented in Supplementary Table 2, were extracted for each of the six time axes making a total of 90 features.

Using random forest classifier as a meta-estimator (34), standard feature selection (FS) methods were applied (35), using a scoring function that measures their “importance” in terms of target predictability [here, Gini impurity; (36)]. After sorting in descending order, redundant variables are identified by their weak contribution to the model accuracy (left y-axis), as shown in Supplementary Figure 5.

After setting a threshold value of 0.025 relative magnitude (right y-axis), the model achieved 95% accuracy, with only twelve most correlated features, thus reducing the curse of dimensionality (37). This threshold value was chosen heuristically using the 'knee of a curve' method, where the optimum point is defined as the accuracy level from which changes in slope (accuracy) are significantly reduced, such that adding further features becomes redundant.

### 3.2. Evaluation

We experimented with several models: Extra trees classifier (ET), AdaBoost (AB), Gaussian Naive Bayes (GNB), gradient boosting (GB), logistic regression (LR), K-nearest neighbors (KNN), support vector machine (SVM), random forest (RF) and decision trees (DT). Table 1 presents a comparison in terms of the evaluation metrics of these models. The best performance of over 95% accuracy was achieved by the KNN method, a non-parametric model which performs classification based on semantic similarity between neighboring data points.

**Table 1:** Model performance comparison between ET, Extra trees classifier; AB, AdaBoost; GNB, Gaussian Naive Bayes; GB, gradient boosting; LR, logistic regression; KNN, K-nearest neighbors; SVM, support vector machine RF, random forest; DT, decision trees (DT).

	**ET**	**AB**	**GNB**	**GB**	**LR**	**KNN**	**SVM**	**RF**	**DT**
Accuracy (%)	94.65	93.10	93.55	95.16	95.02	**95.77**	95.11	93.24	94.48
Precision	0.9383	0.9379	0.9365	0.9462	0.9474	**0.9546**	0.9454	0.9379	0.9502
Recall	0.9392	0.9354	0.9308	0.9428	0.9491	**0.9505**	0.9475	0.9306	0.9423
F_1_-score	0.9468	0.9357	0.9386	0.9479	0.9445	**0.9578**	0.9463	0.9335	0.9482

*The bold value is the best performing model (KNN)*.

## 4. Discussion

Several studies applied smartphone IMUs for gait analysis in the human domain (38–40), and specifically to quantify ataxia (41). However, to the best of our knowledge, our study is the first to show the use of smartphone IMUs to quantify neurological canine gait.

This pilot study showed that inertial data obtained from smartphone IMUs can be used with classical machine learning algorithms to detect ataxia with 95% accuracy. While this accuracy of detection of ataxic gait in dogs is high, further data collection is important to further strengthen the validity of our approach in a clinical setting. These preliminary results highlight the future clinical potential of body-worn smartphone sensors. Only few manipulations of the animal are necessary for analysis, resulting in reduced disturbance of the animals' behavior. In addition, the analysis can be conducted flexibly without increased technical effort on site. Further studies will be needed to investigate potential clinical use of the devices in ataxic dogs.

We have shown here the feasibility of using data obtained by a standard smartphone fixed on the dog's back—a setting that can be easily replicated in any consultation room or home, enabling data acquisition anytime, anywhere. Although large and medium-sized dogs tolerate the placement of smartphone on their backs, smaller dogs might require other solutions. In our study, only 9 dogs with a bodyweight of less than 10 kilogram were included. Safe and stable positioning of the smartphone on top of the dogs' back as well as avoiding artificial alterations of the walking pattern was more challenging in small breeds. For small dogs smartphones might not be the solution, but IMU sensors can be potentially fixed on a collar, or other places on the dog, although it may affect accuracy of detection and requires further research to establish optimal locations.

Using a regular smartphone, with an appropriate recoding application, puts pet owners in a position to contribute in a participatory way to the large-scale collection of data to promote their pet's health. This opens the door to unprecedented opportunities for collecting datasets for gaining understanding into canine neurological disorders in order to enhance detection of pathological alterations. It can also lead to the development of smartphone apps for diagnosing ataxia digitally and remotely. The aspect of domestic data acquisition has another great advantage. Stress in cats and dogs in a clinical setting represents a burden to the animals and the owners, often masking disease and complicating examination and therapy, leading to even more stress (42). Domestic data sampling minimizes stress factors and allows for longitudinal and temporally high-resolution assessment of conditions and its treatments, which add valuable aspects to the on-site visits to the veterinarian.

Here, we have provided a proof of concept study, indicating that the accelerometers and gyroscopes readings obtained from smartphone on a dog's back contain a strong signal related to the characteristics of ataxic gait. One limitation of our study is that we had a limited data set. More data are needed with a higher number of ataxic dogs. It should be noted, that although the KNN algorithm produces good accuracy in our, the classifier is expected to perform slower and costlier in terms of time and memory on larger datasets. However, other simple and more robust algorithms such as SVM performed well on our data, and thus can be also potentially used in future studies. In any case, this provides a starting point for further exploration of larger datasets, which will hopefully lead to digital biomarkers for ataxic gait. Such biomarkers, which are objective, quantifiable data collected by means of digital devices, such as wearables, are increasingly used in human medicine and are typically used to explain, assess and/or predict health-related outcomes (43).

Another limitation is that the low-level statistical features that we used in this study answering if ataxia was present or not, do not provide yet adequate guidance for clinical decision making, nor for monitoring of treatment. Thus, another immediate direction for further studies is exploring the explainability of the resulting models, looking also at individual differences on ataxic patients, and correlating IMU data characteristic with clinical data of the patients.

As initially described, neurological issues can persist for a long time and healing processes can be subtle and long lasting. Extending the presented method from binary classification (ataxic/healthy) to assessing the severity of the ataxia condition will provide a strong basis for a continuous and objective monitoring of the respective condition, probably revealing subtle improvements or deteriorations of ataxic gait. In addition, longitudinal studies about drug impact on ataxic gait patterns could be performed easily.

Finally, in this study we only considered classical machine learning techniques. Obtaining more data samples will allow us to further experiment with deep learning approaches that capture temporal dimensions, which may provide additional insights into the dynamics of ataxic walking patterns.

## 5. Conclusion

Using a simple testing protocol of walking the dogs in a straight line on a leash, we collected IMU data samples from 23 dogs with ataxia, and sampled 55 healthy control group dogs. We experimented with different machine learning classification techniques on the IMU data (3 axes of accelerometer and 3 axes of gyroscope), reaching above 95% accuracy. This indicates potential use for smartphone apps for canine ataxia diagnosis and treatment monitoring in a clinical as well as domestic setting. Future research is needed to optimize and further develop this new diagnostic tool.

## Data Availability Statement

The raw data supporting the conclusions of this article will be made available by the authors, without undue reservation.

## Ethics Statement

The animal study was reviewed and approved by University of Veterinary Medicine Hannover. Written informed consent was obtained from the owners for the participation of their animals in this study.

## Author Contributions

TS, SM, FT, and HV performed data acquisition. TS, SM, FT, HV, and AZ conceived the experiment(s). DE, IK, and AZ ran the experiment and analyzed the data. All authors participated in manuscript writing.

## Funding

The research was partially funded by Israel Ministry of Science (MOST) according to the research project No. 19-57-06007 and by the Israeli Ministry of Agriculture.

## Conflict of Interest

The authors declare that the research was conducted in the absence of any commercial or financial relationships that could be construed as a potential conflict of interest.

## Publisher's Note

All claims expressed in this article are solely those of the authors and do not necessarily represent those of their affiliated organizations, or those of the publisher, the editors and the reviewers. Any product that may be evaluated in this article, or claim that may be made by its manufacturer, is not guaranteed or endorsed by the publisher.
