# Supplementary material for: Measurement of Canine Ataxic Gait Patterns Using Body-Worn Smartphone Sensor Data
Source: Front Vet Sci. 2022 Aug 4;9:912253. doi: 10.3389/fvets.2022.912253 (PMC9386067; doi:10.3389/fvets.2022.912253)
Supplement: Supplementary file 1 [file Data_Sheet_1.pdf]

WS [sec]	Healthy	Ataxic	Ratio	LR Accuracy [%]
7	413	204	2.025	88.651
6	418	198	2.111	91.649
5	409	199	2.055	93.453
4	468	233	2.008	93.981
3.5	713	357	1.997	94.327
**3**	**802**	**408**	**1.966**	**95.023**
2.5	1176	598	1.966	93.836
2	1434	733	1.957	92.224
1.5	1612	828	1.947	88.117
1	2511	1298	1.934	84.314

Table S1: Dataset description: Column 'WS[sec]' presents a fixed window size in seconds; columns 'Healthy' and 'Ataxic' present the number of healthy and ataxic samples obtained for this window size; column 'Ratio' presents Healthy/Ataxic ratio; 'LR Accuracy' presents the logistic regression model accuracy for these WS and samples.

**Domain**	**Features**
Statistical	Mean, mode, standard deviation, median, skewness, kurtosis, Q1, Q3, minimum, maximum, zero-crossing rate (ZCR)
Frequency	Mean, peak, energy, power spectral density (PSD)

Table S2: Extracted Statistical and Frequency Features

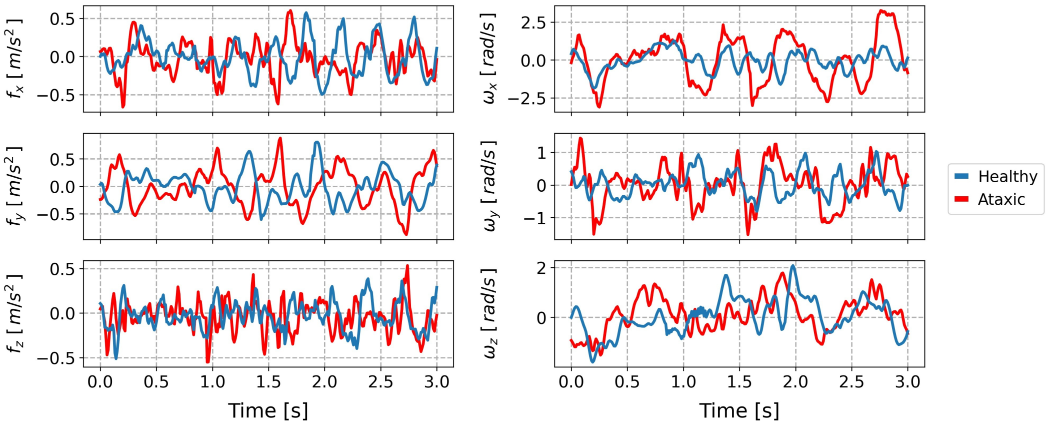

Figure S1: Three seconds window size samples: ataxic (red) vs. healthy (blue) dogs, measured by the accelerometer (left) and the gyroscopes (right).

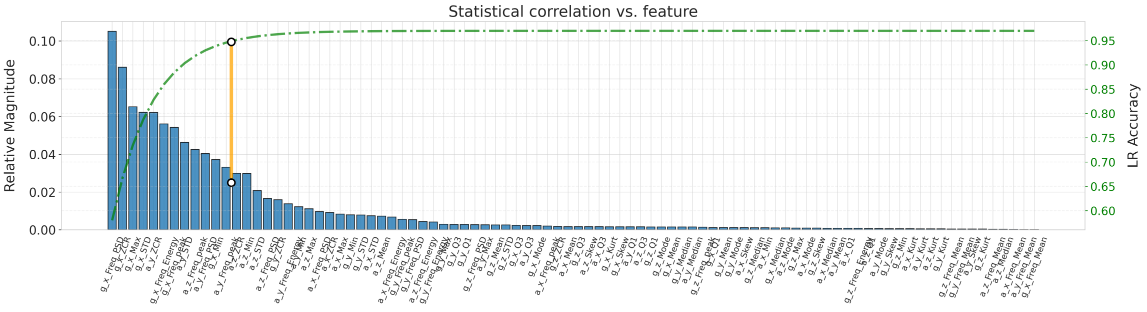

Figure S2: Feature selection process; feature cut-off threshold is marked by the lower bubble.
